# Electrohysterogram for ANN-Based Prediction of Imminent Labor in Women with Threatened Preterm Labor Undergoing Tocolytic Therapy

**DOI:** 10.3390/s20092681

**Published:** 2020-05-08

**Authors:** J. Mas-Cabo, G. Prats-Boluda, J. Garcia-Casado, J. Alberola-Rubio, R. Monfort-Ortiz, C. Martinez-Saez, A. Perales, Y. Ye-Lin

**Affiliations:** 1 Centro de Investigación e Innovación en Bioingeniería, Universitat Politècnica de València, 46022 Valencia, Spain; jmas@ci2b.upv.es (J.M.-C.); gprats@ci2b.upv.es (G.P.-B.); jgarciac@ci2b.upv.es (J.G.-C.); 2 Servicio de Obstetricia, H.U. P. La Fe, 46026 Valencia, Spain; palberola.rubio@gmail.com (J.A.-R.); roger1487@hotmail.com (R.M.-O.); martinez_clasae@gva.es (C.M.-S.); Perales_alf@gva.es (A.P.)

**Keywords:** electrohysterogram, uterine myoelectrical activity, imminent labor prediction, artificial network, tocolytic therapy

## Abstract

Threatened preterm labor (TPL) is the most common cause of hospitalization in the second half of pregnancy and entails high costs for health systems. Currently, no reliable labor proximity prediction techniques are available for clinical use. Regular checks by uterine electrohysterogram (EHG) for predicting preterm labor have been widely studied. The aim of the present study was to assess the feasibility of predicting labor with a 7- and 14-day time horizon in TPL women, who may be under tocolytic treatment, using EHG and/or obstetric data. Based on 140 EHG recordings, artificial neural networks were used to develop prediction models. Non-linear EHG parameters were found to be more reliable than linear for differentiating labor in under and over 7/14 days. Using EHG and obstetric data, the <7- and <14-day labor prediction models achieved an AUC in the test group of 87.1 ± 4.3% and 76.2 ± 5.8%, respectively. These results suggest that EHG can be reliable for predicting imminent labor in TPL women, regardless of the tocolytic therapy stage. This paves the way for the development of diagnostic tools to help obstetricians make better decisions on treatments, hospital stays and admitting TPL women, and can therefore reduce costs and improve maternal and fetal wellbeing.

## 1. Introduction

Preterm birth is defined as delivery prior to the 37^th^ week of gestation [1] and represents between 6%–11% of total births in most European countries, being slightly higher in the U.S. [2]. It is one of the most frequent causes of neonatal deaths [3] and is also related to higher morbidity rates of different health issues, such as neurological disorders in surviving children [1,3]. These children often require close monitoring by specialists during their early years, which entails high costs for families and healthcare systems [3,4]. Some authors report that the cost of a preterm delivery in the US can be five times higher than a term birth [5].

However, the problem lies not only in preterm labor itself but also in threatened preterm labor, which is the most common cause of hospitalization in the second half of pregnancy and involves prolonged stays, more or less aggressive treatment with possible side effects, a cause of worry for the pregnant woman and her family, neglect of other children, etc. Threatened preterm labor also entails high costs, not only for the healthcare system but also due to absence from work. In a study by Lucovnik et al., it was estimated that the average cost of a ’false’ threatened preterm labor was $ 20,372 per patient [6].

Early detection is now the key to preventing preterm labor and mitigating its negative consequences for the newborn. In this regard, tocolytic drugs, which consist of calcium channel blockers, are usually given at the first sign of threatened preterm labor to inhibit uterine contractions and prolong pregnancy as far as possible, while corticosteroids are usually administered at the same time to stimulate fetal maturation [7].

Various labor prediction techniques and measurements have been proposed, such as cervical length, the Bishop score, which evaluates the cervix status, fetal fibronectin, a biochemical marker, and uterine dynamics assessed by tocodynamometry [8,9]. None of these techniques has been shown to objectively and precisely assess time-to-delivery and whether the baby will be premature, mainly due to their low positive predictive values [8,9]. In this respect, uterine electrohysterography (EHG), which records uterine myoelectrical activity on the abdominal surface, has arisen as a powerful tool for preterm labor prediction because of its sensitivity and positive predictive values [8]. It is well known that uterine electrical activity changes throughout pregnancy, and as labor approaches, it becomes more intense and coordinated [10,11], which can be reflected in EHG characteristics. In other words, the signal amplitude increases and spectral content shifts to higher frequencies [12,13,14]. Other authors proposed using non-linear parameters to measure signal regularity, predictability, complexity and/or non-linearity, such as sample entropy [15], the Lyapunov exponent [16], Lempel-Ziv [17], time reversibility [18] for differentiating between preterm labor and term labor and ‘true’ and ‘false’ labor. They found that as pregnancy progresses, the EHG signal non-linearity and predictability increase, while signal complexity is reduced, i.e., the signal patterns are shorter and less varied, although some of these results have been controversial.

Various studies have focused on developing tools and algorithms for predicting labor and/or preterm labor using EHG with very promising results, achieving a predictive model accuracy of over 90% or even 95% [19,20,21,22,23,24,25], although none has had a significant impact on clinical practice, probably because the EHG recordings were conducted on women during regular clinical checkups under physiological conditions. In clinical practice, as tocolytic drugs are usually administered to inhibit uterine contractions at the first sign of threatened preterm labor, EHG recordings may be carried out during different tocolytic therapy phases and produce confusing results [26]. In this respect, in previous work, we found that non-linear parameters such as binary and multistate Lempel-Ziv, spectral entropy and time reversibility could be used for distinguishing women who delivered in over and under 7 days in women undergoing threatened preterm labor [27]. However, the question of predicting labor in these women in different phases of the therapy remains unclear. 

The cross-validation method is commonly used to design and evaluate classifiers [19,20,21,22,24]. A validation dataset is used to fine-tune the model hyperparameters, which may include pruning decision trees, the k value for the nearest neighbor algorithm or learning rate and early stopping of artificial neural networks [28,29]. The performance with new incoming data can be expected to be worse than with those validation datasets. In this regard, it would be advisable to use a testing dataset unfamiliar to the model and cross-validation to further evaluate the classifiers’ generalization capacity and to obtain more realistic model performance indicators for new data.

The aim of this study was thus to develop a generalizable computer-aided system for predicting labor with a 7- and 14-day time horizon in women with threatened preterm labor using temporal, spectral and non-linear parameters to characterize the EHG signals. To determine whether EHG contains enough information to predict labor with these time horizons and whether this information complements obstetric data we developed and compared the model performance with EHG characteristics, obstetric data and a combination of both as input features. The model performance was also assessed with unfamiliar data to test its ability to generalize on new input data.

## 2. Materials and Methods

### 2.1. Database

**Figure 1:**
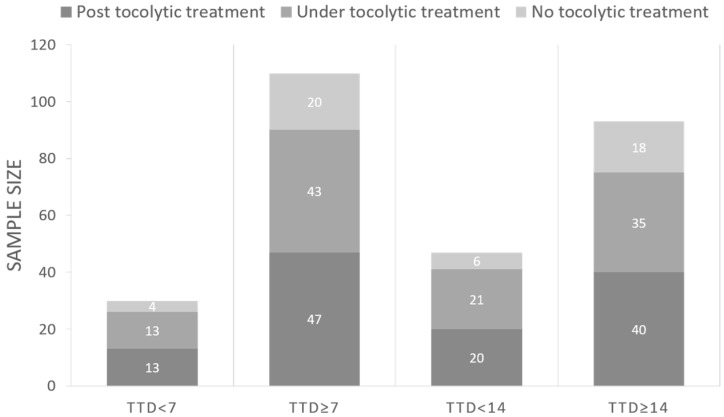
Electrohysterogram (EHG) recording distribution according to time to delivery and tocolytic therapy. TTD: time to delivery.

The database includes 140 30-min EHG recordings from 84 singleton-pregnant women threatened with preterm labor, carried out at the “Hospital Universitari i Politècnic la Fe” (Valencia, Spain) from 2015 to 2019. The study was approved by the hospital´s Institutional Review Board. Patients were informed about the nature of the study and gave their written informed consent. Due to the specific obstetric context, most EHG recordings were taken during or after the administration of Atosiban, which is a widely used inhibitor of uterine contractions. We also collected the normal obstetrical data: maternal age, gestations, parity, abortions, gestational age and cervical length at the recording (six obstetric data). Participants were followed up until delivery, except for women who did not present spontaneous labor. To determine labor predictability with a time horizon of 7 and 14 days, we divided the database into two pairs of groups according to time to delivery (TTD): (1) TTD < 7 days and TTD ≥ 7days, (2) TTD < 14 days and TTD ≥ 14 days. Figure 1 shows the distribution of the number of EHG recordings of the different groups, and also includes the number of recordings taken without, during and after tocolytic therapy in each TTD group. Sample size was 30 and 110 recordings in TTD < 7 and TTD ≥ 7, respectively, and 47 and 93 recordings in TTD < 14 and TTD ≥ 14, respectively.

For the electrode set up, multichannel EHG signals from electrodes arranged to cover the whole uterus can provide detailed information of the underlying uterine dynamics [30]. However, it also increases the recording system complexity and preparation time. To make it easier to use in clinical practice, a single channel was used in the present study. Even though it may not be enough to record weak and isolated uncoordinated contractions, it can be used to estimate time to labor. Two monopolar EHG signals (M1 and M2) were picked up by two disposable Ag/AgCl electrodes (3M red dot 2560, wet with solid hydrogel) on the abdomen 8 cm from each other and symmetrical to the median axis over the navel. Two additional electrodes were placed on each hip as ground and reference electrodes. The signals were digitized with a 24 bit ADC at 500 Hz and down sampled to 20 Hz in the signal preprocessing. One bipolar recording in the 0.1–4 Hz bandwidth was computed as the difference of the two monopolar recordings M1 and M2 to reduce common-mode interferences. Further information on the recording system and data preprocessing can be found in a previous study [27]. Motion-artifacted segments and those with severe respiratory interference were visually identified by experts and discarded.

### 2.2. EHG Characterization

Instead of the traditional EHG-burst analysis, we performed whole EHG window analysis to characterize the EHG signals, since this has been shown to provide relevant information on labor proximity in threatened preterm labor women [27]. The whole window analysis only requires the exclusion of non-physiological segments (motion-artifact segment and respiratory interference) and is easier to integrate in subsequent real-time embedded automatic extraction systems. The EHG characteristics were thus computed in 120 s windows with a 50% overlap to keep representative sections of the recordings at a reasonable computational cost [27]. To obtain a single representative value of each EHG feature for the whole recording and to reduce any redundancy level in the overlap, we computed the median value of all the analyzed windows in the recording.

A set of 23 temporal, spectral and non-linear parameters were computed (see Table 1) in the 0.34–4 Hz fast wave high bandwidth (except for some specific spectral parameters) to characterize the recordings.

As labor approaches, it is expected that EHG signal amplitude increases due to a major recruitment of cell numbers involved in uterine contractions, and that signal spectral content shifts to a higher frequency because of increased cell excitability [31]. Peak-to-peak amplitude, root mean square, interquartile range, integrated EMG, mean absolute value, mean absolute deviation, mean energy and autocorrelation zero crossing have been used to quantify the signal amplitude [19,20]. Since amplitude-related parameters present high interpatient variability and may contain redundant information, we only included peak-to-peak amplitude in this work. Spectral parameters, such as the peak/dominant frequencies [32], median frequency (decile 5) [13] and high-to-low frequency energy ratio (H/L ratio) [33], are often used to assess the spectral content shift to high frequencies associated with labor proximity; these were included in the set of parameters. Power spectrum deciles were also calculated for further characterization of the EHG spectral distribution. Dominant frequency was computed in the range 0.2–1 Hz (DF1) and in 0.34–1 Hz (DF2). Although these frequency bands seem to have considerable overlap, they were defined taking into account the two components of the EHG signal: Fast Wave Low which distributes from 0.2 to 0.34 Hz and Fast Wave High, which comprises components above 0.34 Hz [10,34]. The H/L ratio was thus computed as the ratio of energy in 0.34–1 Hz to energy in 0.2–0.34 Hz. Teager energy was also calculated, since it not only contains information on signal amplitude, but also on the frequency content and it has been related to uterine contraction intensity [35].

As biological processes are known to be highly non-linear, a set of non-linear parameters which had previously been used to characterize the EHG signal were also computed. We included sample [15] and fuzzy entropy [36] to measure time series complexity based on the self-similarity of different subsequences in time, and Lempel-Ziv (Binary and multistate n = 6) [37], which evaluates signal complexity from the number of different patterns in the time series. Spectral entropy [38], which estimates signal complexity by analyzing the time series in the frequency domain, and time reversibility [16], which estimates the similarity of a time series when time goes forward or back, were also computed. We did not include any other non-linear parameters that had previously been used to discriminate term and preterm labor such as the Lyapunov exponent [16], due to its poor predictive value, or approximate and Shannon entropy [22,23], due to their having mutual and/or redundant information.

Since the “present” EHG signal amplitude significantly influences the “following” values [39], a Poincaré plot of consecutive EHG signal samples was obtained by representing EHG[n] with respect to EHG[n-1] in each analysis window to estimate the short- and long-term variation. SD1 and SD2 were also computed. These represent the dispersion along the minor and major axes of the ellipse in the Poincaré plot [39], which can been associated with short and long term EHG signal variation, respectively. We also computed the SD1/SD2 ratio, which is a measure of signal randomness [40].

Wilcoxon rank sum test was used to check for any statistically significant differences between the groups (TTD < 7 vs. TTD ≥ 7, and TTD < 14 vs. TTD ≥ 14) for each obstetric and EHG parameter.

### 2.3. Classifiers Design and Evaluation

We attempted to determine whether EHG contained relevant labor prediction information for a 7- and 14-day time horizon in threatened preterm labor women and whether this information complemented the obstetric data. For this, we designed and tested three different classifiers with different input labor prediction features: EHG characteristics only, obstetric data only and a combination of EHG characteristics and obstetric data.

As can be seen in Figure 1, the available data set was clearly imbalanced between classes in all the time horizons (N = 30 TTD < 7 vs. 110 TTD ≥ 7 and N = 43 TTD < 14 vs. 97 TTD ≥ 14). This entailed a higher risk of detecting the majority class at the expense of the minority. To deal with this issue, we oversampled the minority classes by the synthetic minority oversampling technique (SMOTE) [41], which has been widely used to solve imbalanced class problems [19,22,33,42]. Five neighbors were used to interpolate new cases in the minority class, as in a previous work [33]. The conventional holdout method was then used to train and validate the classifiers (30 partitions). To determine the model’s generalization capacity, we further evaluated its performance by new testing data ‘unseen’ by the model. For each partition, we randomly split the whole balanced database into three datasets with the same proportion between classes in each: training (1/3), validation (1/3) and testing (1/3).

Given the sample size of the database and the number of input features (23 EHG characteristics and/or six obstetric data), dimension reduction techniques were necessary to mitigate possible overfitting problems when designing the classifier. A principal component analysis was performed to retain a relatively high amount of the variance (98%), while significantly reducing the number of input features [25,36,43].

We considered the most common types of classifiers for forecasting applications such as k-nearest neighbor (KNN), random forest, support vector machines (SVMs) and artificial neural networks (ANNs), [44,45,46,47]. KNN classifiers often present lower metric values than SVM and ANN on relatively small datasets [44,47]. Additionally, KNN greatly depends on the training set, since these classifiers are generated from the training samples only and do not use any additional data, which may reduce its generalization capacity [48]. On the other hand, both SVM and ANN are universal function approximates, although they may present differences when classifying non-linear data. ANN consists of multilayer connections with several activation functions to deal with nonlinear problems [44]. SVM makes use of nonlinear mapping to make data separable. In this regard, the selection of the kernel on classifier performance is a critical factor [45,47,49]. SVM and ANN generally show a relatively similar performance, while ANNs are slightly more accurate in classification problems [49]. ANNs have also been widely used in preterm labor prediction applications [19,23]. Peng et al. used random forest based on EHG features for predicting preterm labor, although the performance was not quite as good as ANN [22]. We therefore chose ANN to implement the classifiers in the present work.

**Figure 2:**
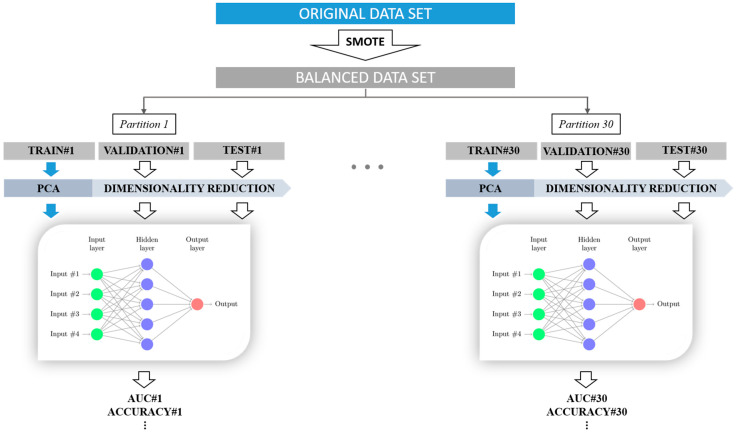
Diagram of the method used to train and validate the labor prediction model with 7- and 14-day time horizons in threatened preterm labor women.

A multilayer perceptron structure (MLP) was applied with the hyperbolic tangent as activation function. The structure consisted of three layers of neurons: input layer, hidden layer and output layer [50]. The ANN weights were set iteratively using the widely used backpropagation algorithm [51]. We carried out a grid search of the number of neurons in the hidden layer, from 2 to 10 for each classifier, to achieve the optimal ANN topology. The maximum number of neurons was limited to 10 since a large number of hidden units may lead to overfitting [52]. The “early stopping” technique was applied for regularization, since it has been reported to significantly reduce overfitting, in combination with the backpropagation algorithm [51,52]. To obtain truly generalizable classifiers, the optimal topology was finally selected according to the training and validation dataset’s performance, retaining the “unseen” testing data. Figure 2 shows the method used to design and validate the threatened preterm labor prediction model.

A set of metrics was obtained for each partition to evaluate the model’s performance in training, validation and testing the data: accuracy, area under the ROC curve (AUC), F1-score, sensitivity, specificity, predictive positive value (PPV) and predictive negative value (PNV):

(1) $$Accuracy\left(\%\right)=\frac{TP+TN}{\left(TP+TN+FP+FN\right)}$$

(2) $$Sensibility\;\left(\%\right)=\frac{TP}{\left(TP+FN\right)}$$

(3) $$Specificity\;\left(\%\right)=\frac{TN}{\left(TN+FP\right)}$$

(4) $$PPV\;\left(\%\right)=\frac{TP}{\left(TP+FP\right)}$$

(5) $$PNV\;\left(\%\right)=\frac{TN}{\left(TN+FN\right)}$$

(6) $$F1\text{-}score=\frac{2*TP}{2*TP+FP+FN}$$

where, TP, TN, FP and FN are the acronyms of true positives, true negatives, false positives and false negatives, respectively. In this application a true positive is labor TTD < 7 days or TTD < 14 days, according to the classifier criteria.

All the signal processing algorithms were developed on Matlab 2017a on Windows 10 operating system. Sample entropy, fuzzy entropy and Lempel-Ziv EHG parameters as well as the generation of synthetic data for minority class with SMOTE were computed on free open-code. The computation code for the remaining features was implemented by the research group. The ANN classifier was developed on Matlab Statistics and Machine Learning Toolbox.

## 3. Results

For both time horizons, mean and SD values of obstetric data are given in Table 2 and mean and SD of EHG characteristics in Table 3. As expected, cervical length decreased as labor approached, with statistically significant differences between TTD < 7 vs. TTD ≥ 7 and TTD < 14 vs. TTD ≥ 14. Gestational age at recording was higher for the lower TTD group, with statistically significant differences only between women who delivered in less and more than 7 days. No significant differences were found in the remaining obstetric data (maternal age, gestation, parity and abortion).

EHG characteristics of peak-to-peak amplitude, spectral parameters (DF1, DF2, H/L ratio, Deciles) and Teager energy were slightly, but not significantly, lower in TTD < 7 and TTD < 14 groups than TTD ≥ 7 and TTD ≥ 14, respectively. Except for sample entropy, the non-linear parameters extracted from TTD < 7 (binary Lempel-Ziv, multistate Lempel-Ziv, spectral entropy and fuzzy entropy), which assess the degree of signal complexity, were significantly lower than TTD ≥ 7. However, no statistically significant differences were found in these parameters between <14 and ≥14-day deliveries. In contrast, the time reversibility of EHG recordings increased as labor approached, obtaining statistically significant differences between the two delivery groups in both 7- and 14-day time horizons. Of the parameters derived from the Poincaré plot, only the SD1/SD2 ratio obtained significant differences between TTD < 7 and TTD ≥ 7 groups.

**Figure 3:**
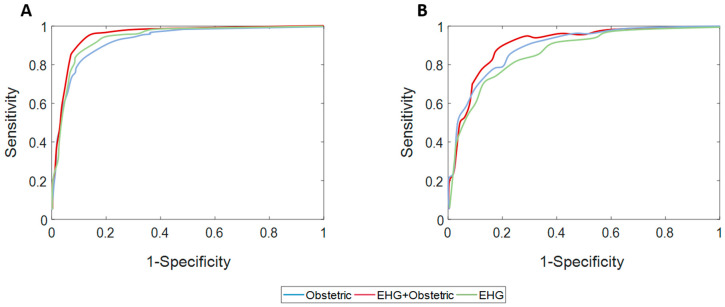
Average ROC curves for testing data when using different input features to predict labor in <7 days (**A**) and <14 days (**B**).

**Figure 4:**
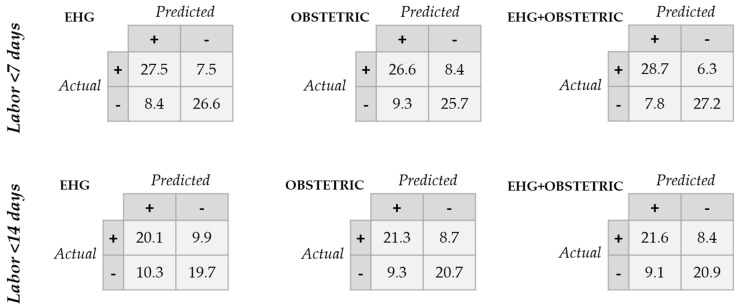
Average confusion matrices for testing data when using different input features to predict labor in <7 days and <14 days.

Table 4 and Table 5 show the performance indicators for model training, validation and testing data in predicting labor with 7- and 14-day time horizons, respectively. Figure 3 gives the average testing data ROC curve when using different sets of input labor prediction features. Figure 4 contains the average confusion matrixes for the different classifiers when evaluated in the testing data. In general, the model’s training data performance was better than for validation data, which in turn outperformed testing data. In labor prediction with a 7-day time horizon, the greatest difference between training and testing data was obtained for obstetric data, suggesting a certain degree of overfitting. The predictive model with EHG characteristics outperformed that obtained from obstetric data for predicting labor in less than 7 days (average AUC: 84.4 ± 4.8% for EHG characteristics vs. 79.6 ± 6.3% for obstetric data). The best result was obtained when combining EHG characteristics and obstetric data as the model input feature, achieving an AUC of 87.1 ± 4.3%, accuracy of 80.2 ± 4.5%, and F1-score of 80.3 ± 5.5% (see Figure 3). The model performance deviation between different data partitions was relatively small, suggesting that the method is robust against random dataset variability.

Nevertheless, when attempting to predict labor in <14 days, the average AUC of the predictive model using EHG declined below 72% in the test data, which is even less than that obtained using obstetric data inputs. This phenomenon can also be seen in Figure 3. When both EHG characteristics and obstetric data are the input features, the model performance barely improves, with an AUC of 76.2 ± 5.8%, an accuracy of 71.1 ± 5.7% and F1-score of 70.8 ± 6.9%. Model performance presented a greater deviation between the partitions when compared with 7-day labor prediction.

## 4. Discussion

The aim of this study was to develop a labor prediction system with time horizon of 7 and 14 days in women with threatened preterm labor based on EHG parameters and to compare the model’s performance with that of obstetrical data. Firstly, we analyzed the discriminatory capability of temporal, spectral and non-linear parameters computed from EHG recordings to differentiate TTD < 7 and TTD < 14 from TTD ≥ 7 and TTD ≥ 14 groups, respectively. Previous studies computed similar parameters from intrinsic mode functions after applying empirical mode decomposition [22] or wavelet packet decomposition [20] for predicting preterm labor, which considerably increases the computational cost, and this can be a key factor in transferring the EHG technique to clinical practice. We therefore preferred to compute them from ‘raw signals’ considering different bandwidths when necessary. To facilitate clinical use, we also simplified the recording protocol by only acquiring one bipolar EHG signal, so that it was not feasible to compute multivariate parameters and synchronization indexes [36,53].

It is well known that as labor approaches, EHG signal amplitude increases due to a major recruitment of cell numbers involved in uterine contractions, and that signal spectral content shifts to a higher frequency because of increased cell excitability [31]. Most et al. found significant differences in RMS values between deliveries in more or less than 14 days in healthy women in drug-free condition [54]. It has been reported that dominant frequency computed in the 0.24–4 Hz frequency range in threatened preterm labor women who delivered in less than 7 days and did not receive any tocolytic therapy was significantly higher than those who delivered after 7 days [17]. Nevertheless, we did not find significant differences in peak-to-peak amplitude nor in spectral parameters associated to ‘imminent’ delivery in our database. This is in agreement with our previous study with a smaller database (N = 88), in which we compared a smaller set of amplitude and spectral EHG features from women under tocolytic treatment delivering in less and more than 7 days. [27].

This disagreement with the state of the art could be attributed to several factors. Firstly, amplitude-related parameters are not reliable indicators of labor proximity [13] due to their high inter-patient variability and the strong influence of other factors such as skin preparation and inter-electrode distance. Secondly, spectral parameters such as dominant frequency do not follow a monotonous increasing trend with TTD [31]. Maner et al. found a slight increase of up to TTD = 20 days, followed by a steeper drop for TTD = 10 and an abrupt rise for TTD = 1 [31]. The time horizons used in the present study (7 and 14 days) are in this range, in which the spectral parameters may be reduced. Moreover, most of the present EHG recordings (≈84%) were taken from patients either undergoing or who had received tocolytic treatment. In this respect, tocolytic drugs such as Atosiban, which is a calcium channel blocker commonly used to inhibit uterine contractions, has been proven to shift signal spectral content to lower frequencies—especially in women closer to delivery—thereby reducing PSD peak frequency and/or the high-to-low frequency content ratio [55]. We consider that these factors could be the main reason for the disagreement between our present results with others in the literature.

On the other hand, non-linear parameters were found to be more reliable indicators of the proximity of delivery, obtaining significance differences for differentiating women who delivered under and over 7 days, which is consistent with our previous study [27]. Sample entropy, fuzzy entropy and spectral entropy decreases as labor approaches, due to higher signal regularity and predictability, which is in agreement with FeleZorz [12]. However, we found that Lempel-Ziv decreases as labor approaches, which disagrees with Lemancewicz et al., who obtained higher values for threatened preterm labor women who delivered in <7 days [17]. We attribute this possible discrepancy to specific computational factors, such as different bandwidth, analysis window length and a possible difference on inclusion/exclusion criteria of the EHG signal segment affected by motion artifacts or respiratory interference. The different drugs used may also contribute to this discrepancy.

In this work, we found that only time reversibility provides a significant difference for women who delivered in ±7 and ±14 days, which agrees with other studies that found that time reversibility outperformed sample entropy and spectral parameters for predicting labor [16]. No significant differences were found for discriminating the TTD < 7/14 and TTD ≥ 7/14 group in either SD1 or SD2 for the parameters derived from the Poincaré plot. We believe that the tocolytic drugs affected the discriminatory capability of these labor prediction parameters, since they have been correlated with high frequency power, and both low and high frequency powers, respectively [40]. Nevertheless, we found that the SD1/SD2 ratio was less influenced by the tocolytic drug, and a significantly lower value was obtained for women who delivered in less than 7 days, suggesting that EHG signal randomness is reduced as labor approaches.

We also reported and compared for the first time the prediction system performance for detecting labor with a 7-day time horizon in threatened preterm labor women using EHG characteristics and/or obstetrical data. The main contribution to the state of the art was the specific obstetric context of the EHG recordings: women with threatened preterm labor who may have been before, under or after tocolytic treatment. In this regard, most published studies focus on the feasibility of predicting preterm labor in women during regular checkups, when EHG is recorded in a drug-free physiological state [19,20,21,22,32,33,36,56]. Firstly, the AUC of our model for validation data was higher than 90%, which is comparable with those reported by other authors who attempted to predict preterm labor during regular checkups [19,20,21,22,32,36,56]. Unlike the studies in which the model was validated by the 10 k-fold or holdout cross validation method, we further proved the model’s generalization capability on testing data that had never been seen by the model. The AUC for predicting labor in less than 7 days was of 87.1 ± 4.3% (test dataset), which can be considered a more realistic expected performance for predicting imminent labor for the new incoming data. The result achieved in the present work is slightly inferior to the model performance for testing data for predicting preterm labor in women in regular checkups by EHG (AUC~91%) [33], which can be attributed to the influence of the tocolytic drug on the EHG signal [26,55].

In addition, EHG outperformed obstetric data for predicting imminent labor (in less than 7 days), with cervical length being the most significant data. We believe that our model also outperforms the logistic regression model, which achieved a c-statistic of 89% (without validation and test data), using maternal age, vaginal bleeding, cervical length and fetal fibronectin for predicting labor in less than 7 days after a successful 48-h treatment with threatened preterm labor [57]. Our results indicate that regardless of the tocolytic treatment phase at the EHG recording, EHG contains relevant information on the uterine electrophysiological status and other complementary information to predict imminent labor. This finding agrees with other authors, who found that the labor mechanism may be initiated days or weeks before preterm labor [58]. The development of an imminent labor prediction system would allow obstetricians to make better decisions regarding treatments, hospital stays and admission of threatened preterm labor women, thereby reducing costs and improving maternal and fetal wellbeing.

In contrast, the model performance in predicting labor in less than 14 days using the EHG characteristics was relatively low, with an AUC of 71.1 ± 7.4%, which could be due to EHG containing relatively less information with this time horizon. Indeed, our results suggest that EHG contains less information than obstetric data for predicting labor in less than this time, obtaining lower model performance (see Table 5 and Figure 3B). As previously stated, only time reversibility provided a significant difference for discriminating women who delivered in less and more than 14 days. This impairment of the model performance with respect to that of predicting imminent labor in 7 days may be explained by the fact that the electrophysiological changes associated with labor have not yet taken place in this time horizon. In this respect, the prediction of preterm labor with a still longer time horizon by EHG recordings in threatened preterm labor women undergoing tocolytic treatment could be even more challenging.

To sum up, the results for the first time proved the feasibility of imminent labor prediction by EHG in women with threatened preterm labor undergoing tocolytic treatment, which is potentially highly likely to become clinical practice. However, despite its promising results, the present study is not exempt from limitations. Firstly, the current database is relatively small and may have a limited capacity to represent the target population, especially the TTD < 7 group. However, we were able to record 10 new cases for this group and no significant differences were obtained when compared with the 30 EHG recordings included here. A larger database is still needed to further validate the performance of the imminent labor prediction system. Secondly, the EHG recordings were taken from women who could be either before, undergoing or after tocolytic treatment, and this could be an important confounding prediction factor. In this regard, the contextualization of EHG characteristics taking the tocolytic treatment stage into account, or the development of specific classifiers for each drug treatment stage could enhance the imminent labor prediction system performance. Thirdly, the sample size of the women delivering <7 days was about 25% smaller than ≥ 7 days, giving rise to the problem of inter-class data imbalance. We here mitigated this problem by means of the commonly-used SMOTE oversampling technique. Specific imbalanced data learning algorithms, such as weighted classifiers or boosting ensemble learning, could contribute to achieving more reliable imminent labor prediction systems. 

We also consider that it will be necessary to develop other signal processing techniques and/or features to predict delivery in less than 14 days to enhance the current prediction system performance as regards the transfer to clinical practice. It should be noted that if the EHG-based imminent labor prediction system is to be used in clinics, it would also be necessary to include the automatic exclusion of artifacted signal segments and EHG feature extraction to feed the classifier. Although some preliminary work has been done on this [58], it is still one of the most difficult challenges remaining.

## 5. Conclusions

We analyzed the discriminatory capability of EHG characteristics for distinguishing labor with time horizons of 7 and 14 days in women with threatened preterm labor. Due to the confusing tocolytic drug factor, which has been proven to reduce cell excitability and shift signal spectral content to lower frequencies, the discriminatory capacity of the EHG temporal and spectral parameters was somewhat limited. However, non-linear parameters were found to be more reliable indicators of labor proximity in threatened preterm labor women, with binary and multistate Lempel-Ziv, fuzzy entropy, spectral entropy and SD1/SD2 ratio being significantly lower for the TTD < 7 than the TTD ≥ 7 group. Time reversibility is the only EHG-related parameter which has been shown to provide significant differences between women who delivered in ± 7 and ± 14 days. 

We also developed expert systems for predicting labor in 7 and 14 days in women with threatened preterm labor. Regardless of the input features (EHG, obstetric data or all together) used to train the model for predicting labor in 14 days, model performance was moderate (AUC < 76%). This could have been due to the fact that the electrophysiological changes associated with labor had not yet taken place. However, our results did show that EHG contains relevant information on the uterine electrophysiological status to complement obstetric data for predicting imminent labor in less than 7 days in threatened preterm labor women. When using both EHG characteristics and obstetric data as input features, the AUC for predicting labor in less than 7 days was 87.1 ± 4.3% for testing data that had not been previously seen by the model. These prediction systems could allow better attention to real imminent labor to mitigate the negative consequences of preterm delivery and improve maternal and fetal wellbeing. In longer TTD horizons, the prediction system could help to optimize hospital resources and significantly reduce costs.

## Figures and Tables

**Table 1:** Summary of different EHG features and obstetric data.

EHG Temporal Parameters	EHG Spectral Parameters	EHG Non-Linear Parameters	Obstetric Data
Peak-to-peak amplitude	DF1 DF2 H/L Ratio Deciles [D1-D9] Teager Energy	Binary Lempel-Ziv Multistate Lempel-Ziv (n = 6) Sample Entropy Spectral Entropy Fuzzy Entropy Time reversibility SD1 SD2 SD1/SD2	Cervical length Gestational age at moment of recording Maternal age Gestations Parity Abortions

**Table 2:** Mean and SD of obstetric data of women who delivered in less and more than 7 days and 14 days. *p*-value < 0.05 (grey shading) indicates significant differences between both groups.

	TTD < 7	TTD ≥ 7	*p*-Value	TTD < 14	TTD ≥ 14	*p*-Value
**Cervical length (mm)**	13.67 ± 8.32	21.34 ± 12.07	0.001	14.13 ± 8.67	22.59 ± 12.17	7 × 10^−5^
**Gestational age at recording (weeks)**	32.17 ± 2.09	30.50 ± 3.10	0.003	31.48 ± 2.34	30.53 ± 3.23	0.075
**Maternal age (years)**	31.80 ± 4.56	31.89 ± 6.17	0.628	31.73 ± 5.66	31.95 ± 5.97	0.937
**Gestation**	1.80 ± 1.27	1.85 ± 1.13	0.487	1.69 ± 1.11	1.91 ± 1.18	0.140
**Parity**	0.40 ± 0.67	0.45 ± 0.58	0.481	0.39 ± 0.58	0.52 ± 0.60	0.065
**Abortion**	0.27 ± 0.83	0.33 ± 0.69	0.196	0.31 ± 0.80	0.32 ± 0.68	0.386

**Table 3:** Mean and SD of EHG characteristics of 7- and 14-day women. *p*-value < 0.05 (grey shading) indicates significant differences between both groups.

	TTD < 7	TTD ≥ 7	*p*-Value	TTD < 14	TTD ≥ 14	*p*-Value
Peak-to-peak amplitude (µV)	144.0 ± 72.4	154.8 ± 206.5	0.482	136.4 ± 63.5	160.8 ± 224.5	0.977
DF1 (Hz)	0.269 ± 0.02	0.266 ± 0.019	0.650	0.268 ± 0.02	0.266 ± 0.019	0.520
DF2 (Hz)	0.399 ± 0.015	0.401 ± 0.024	0.966	0.397 ± 0.014	0.403 ± 0.025	0.374
H/L Ratio	0.410 ± 0.084	0.428 ± 0.073	0.237	0.42 ± 0.09	0.426 ± 0.068	0.344
Decile 1 (Hz)	0.223 ± 0.007	0.223 ± 0.006	0.976	0.224 ± 0.008	0.222 ± 0.005	0.460
Decile 2 (Hz)	0.243 ± 0.012	0.243 ± 0.011	0.579	0.245 ± 0.015	0.242 ± 0.009	0.797
Decile 3 (Hz)	0.262 ± 0.016	0.264 ± 0.014	0.509	0.265 ± 0.018	0.263 ± 0.012	0.642
Decile 4 (Hz)	0.284 ± 0.019	0.287 ± 0.017	0.369	0.287 ± 0.02	0.286 ± 0.016	0.974
Decile 5 (Hz)	0.308 ± 0.023	0.312 ± 0.019	0.383	0.311 ± 0.022	0.312 ± 0.018	0.781
Decile 6 (Hz)	0.336 ± 0.028	0.341 ± 0.022	0.271	0.338 ± 0.025	0.341 ± 0.022	0.445
Decile 7 (Hz)	0.371 ± 0.032	0.380 ± 0.026	0.109	0.374 ± 0.028	0.381 ± 0.027	0.177
Decile 8 (Hz)	0.427 ± 0.039	0.439 ± 0.033	0.077	0.431 ± 0.037	0.439 ± 0.034	0.161
Decile 9 (Hz)	0.525 ± 0.045	0.541 ± 0.038	0.103	0.530 ± 0.044	0.541 ± 0.038	0.221
Teager energy (a.u.)	8.6 ± 8.8	21.6 ± 19.9	0.183	9.1 ± 8.6	23.9 ± 19.8	0.440
Binary Lempel-Ziv	0.388 ± 0.066	0.437 ± 0.075	0.002	0.411 ± 0.075	0.435 ± 0.075	0.062
Multistate Lempel-Ziv	0.210 ± 0.058	0.241 ± 0.062	0.011	0.231 ± 0.062	0.236 ± 0.063	0.510
Sample entropy	2.173 ± 0.308	2.272 ± 0.243	0.143	2.261 ± 0.27	2.245 ± 0.257	0.158
Spectral entropy	0.874 ± 0.018	0.887 ± 0.022	0.003	0.881 ± 0.02	0.886 ± 0.022	0.078
Fuzzy entropy	0.264 ± 0.06	0.308 ± 0.064	0.002	0.287 ± 0.065	0.304 ± 0.066	0.152
Time reversibility	4.858 ± 3.182	3.554 ± 2.049	0.011	4.538 ± 2.649	3.467 ± 2.161	0.001
SD1	2.86 ± 1.62	3.54 ± 3.18	0.126	3.038 ± 1.527	3.59 ± 3.434	0.385
SD2	26.28 ± 13.03	27.02 ± 25.19	0.501	25.28 ± 11.57	27.68 ± 27.24	0.809
SD1/SD2	0.116 ± 0.032	0.141 ± 0.045	0.003	0.129 ± 0.04	0.14 ± 0.045	0.119

**Table 4:** Mean and SD of model performance indicators in predicting labor in under or over 7 days using EHG characteristics, obstetric data or a combination of both.

	EHG			OBSTETRIC			EHG+OBSTETRIC		
	**Train**	**Validation**	**Test**	**Train**	**Validation**	**Test**	**Train**	**Validation**	**Test**
**Accuracy**	88.2 ± 4.4	82.6 ± 5.8	76.8 ± 4.1	93.0 ± 4.9	90.9 ± 5.5	74.9 ± 5.2	89.9 ± 4.3	84.0 ± 4.9	80.2 ± 4.5
**AUC**	92.7 ± 3.7	90.0 ± 4.3	84.4 ± 4.8	94.9 ± 3.9	93.8 ± 4.1	79.6 ± 6.3	94.5 ± 3.1	91.8 ± 3.2	87.1 ± 4.3
**F1-Score**	88.3 ± 4.3	82.9 ± 6.0	77.1 ± 5.1	93.0 ± 4.9	91.1 ± 5.2	75.4 ± 5.4	89.9 ± 4.2	84.3 ± 5.0	80.3 ± 5.5
**Sensibility**	89.0 ± 5.2	84.8 ± 9.1	79.4 ± 9.7	94.1 ± 5.6	93.0 ± 6.1	77.7 ± 7.7	90.0 ± 4.8	86.5 ± 7.4	81.6 ± 9.4
**Specificity**	87.4 ± 5.9	80.4 ± 8.9	74.1 ± 8.3	91.8 ± 5.2	88.8 ± 7.6	72.1 ± 6.2	89.8 ± 6.5	81.5 ± 7.3	78.8 ± 5.8
**PPV**	87.8 ± 5.1	81.7 ± 7.0	75.9 ± 4.8	92.0 ± 4.9	89.6 ± 6.6	73.6 ± 4.9	90.1 ± 5.7	82.7 ± 5.5	79.6 ± 4.1
**NPV**	88.9 ± 4.8	84.8 ± 7.9	79.1 ± 6.4	94.1 ± 5.7	92.9 ± 6.0	76.7 ± 6.7	90.1 ± 4.6	86.2 ± 6.3	81.8 ± 7.0

**Table 5:** Mean and SD of model performance indicators in predicting labor in under or over 14 days using EHG characteristics, obstetric data or a combination of both. PPV: predictive positive value; NPV: predictive negative value.

	EHG			OBSTETRIC			EHG+OBSTETRIC		
	**Train**	**Validation**	**Test**	**Train**	**Validation**	**Test**	**Train**	**Validation**	**Test**
**Accuracy**	81.2 ± 7.0	75.4 ± 7.1	65.6 ± 5.6	81.3 ± 11.5	75.8 ± 11.5	70.4 ± 8.2	83.6 ± 3.7	78.9 ± 3.7	71.1 ± 5.7
**AUC**	86.4 ± 7.8	83.6 ± 7.3	71.1 ± 7.4	83.8 ± 10.7	81.8 ± 11.1	75.5 ± 8.2	89.5 ± 3.2	86.6 ± 3.1	76.2 ± 5.8
**F1-Score**	81.7 ± 4.9	75.3 ± 5.6	65.9 ± 5.7	82.1 ± 10.5	75.8 ± 11.8	70.8 ± 8.3	83.5 ± 3.9	76.5 ± 4.3	70.8 ± 6.9
**Sensibility**	83.0 ± 6.6	74.8 ± 11.3	67.6 ± 13.2	85 ± 12.2	77.1 ± 16.1	72.6 ± 12.9	87.6 ± 6.0	77.5 ± 8.7	70.8 ± 12
**Specificity**	79.3 ± 16.1	76.0 ± 14.2	63.6 ± 12.6	77.7 ± 17.9	74.4 ± 18.8	68.2 ± 17.0	83.7 ± 5.3	78.7 ± 9.3	70.0 ± 10.9
**PPV**	81.3 ± 4.6	78.4 ± 10.0	66.5 ± 6.4	80.2 ± 11.1	76.4 ± 11.8	70.6 ± 8.9	83.8 ± 4.5	78.8 ± 6.6	68.8 ± 6.9
**NPV**	95.5 ± 4.5	90.8 ± 5.2	62.9 ± 6.9	90.0 ± 8.4	88.8 ± 10.0	65.5 ± 7.4	83.8 ± 5.1	76.7 ± 5.0	68.8 ± 7.7
